# Supplementary material for: Biophysical characterization of the ETV6 PNT domain polymerization interfaces
Source: J Biol Chem. 2021 Jan 13;296:100284. doi: 10.1016/j.jbc.2021.100284 (PMC7949025; doi:10.1016/j.jbc.2021.100284)
Supplement: Figures and Tables [file mmc1.pdf]

Supporting Information

Biophysical characterization of the ETV6 PNT domain polymerization interfaces

Chloe A. N. Gerak[1], Sophia Y. Cho[1], Maxim Kolesnikov[2], Mark Okon[1], Michael E. P. Murphy[2], Richard B. Sessions[3], Michel Roberge[1], Lawrence P. McIntosh[1,4*]

[1]Department of Biochemistry and Molecular Biology, University of British Columbia, Vancouver, BC, Canada

[2]Department of Microbiology and Immunology, University of British Columbia, Vancouver, BC, Canada

[3]School of Biochemistry, University of Bristol, Bristol, United Kingdom

[4]Department of Chemistry, University of British Columbia, Vancouver, BC, Canada

***Corresponding author** Lawrence P. McIntosh

Email: mcintosh@chem.ubc.ca

Running title: Characterizing the ETV6 PNT domain interaction

Table S1. Protein sequences.

Sequence Description	Sequence
**His$_6$-tagged ETV6$^{1\text{-}125}$**<br>The His$_6$-tag (green) precedes a thrombin cleavage site (/) and the wild type starting Met residue for ETV6 (yellow highlight). The second thrombin cleavage site (//) and alternative start site (M43) are also indicated. The structured PNT domain spans residues L50-Q123 (magenta). Monomerizing mutations were introduced at A93 (cyan highlight) and V112 (green highlight).	MGSSHHHHHHSSGLVPR/GSHMMSETPAQCSIKQERISYTPPESPVPSYASSTPLHVPVPR//ALRMEEDSIRLPAHLRLQPIYWSRDDVAQWLKWAENEFSLRPIDSNTFEMNGKALLLLTKEDFRYRSPHSGDVLYELLQHILKQRK
**His$_6$-tagged, Avitag-ETV6$^{43\text{-}125}$**<br>The His$_6$-tag (green) precedes a thrombin cleavage site (/) and the Avitag (black highlight) with M43 (yellow highlight) as the start site. The structured PNT domain spans residues L50-Q123 (magenta). Monomerizing mutations were introduced at A93 (cyan highlight) and V112 (green highlight).	MGSSHHHHHHSSGLVPR/GSHIGLNDIFEAQKIEWHEHMEEDSIRLPAHLRLQPIYWSRDDVAQWLKWAENEFSLRPIDSNTFEMNGKALLLLTKEDFRYRSPHSGDVLYELLQHILKQRK

Table S2. Data collection and refinement statistics for the ETV6 A93D-V112E-PNT domain.

**Data collection**	
Space group	$P6_522$
Cell dimensions	
a, b, c (Å)	59.9, 59.9, 169.4
α, β, γ (°)	90, 90, 120
Resolution range (Å)	44.2 – 1.9 (1.92 – 1.85)
R-merge	0.077 (1.29)
$I / \sigma I$	2.6 (2.8)
CC(1/2) (%)	99.9 (90.1)
Wilson B (Å^2)	30.2
Completeness (%)	100.0 (99.9)
**Refinement**	
Resolution (Å)	1.85
No. unique reflections	16166 (1576)
R_{work}	0.200 (0.234)
R_{free}	0.234 (0.300)
No. of non-hydrogen atoms	1439
Macromolecules	1328
Ligands	6
Solvent	105
Average B-factor (Å^2)	33.7
Macromolecules	33.3
Ligands	39.6
Solvent	38.6
RMS deviations	
Bond lengths (Å)	0.006
Bond angles (°)	0.73
Ramachandran favored (%)	99.3
Ramachandran allowed (%)	0.67
Ramachandran outliers (%)	0.00
Rotamer outliers (%)	0.69

(Statistics for the highest-resolution shell are shown in parentheses)

Table S3. Amide HX protection factors.

Residue	A93D-PNT Monomer log(PF) [a]	V112E-PNT Monomer log(PF) [a]	A93D-PNT Heterodimer log(PF) [a]	V112E-PNT Heterodimer log(PF) [a]	amide $^1H^N$ h-bond acceptor [b]	2º struct. [b]
L41						
R42						
M43						
E44						
E45						
D46						
S47						
I48						
R49						
L50						
P51						
A52						3_{10}
H53						3_{10}
L54					P51 CO	3_{10}
R55					A52 CO	
L56						
Q57						
P58						3_{10}
I59					Q57 CO	3_{10}
Y60	4.82	4.72	5.17	5.35	Q57 CO	3_{10}
W61	5.47	5.29	6.16	6.39	P58 CO	
S62						
R63						α
D64						α
D65	3.78	3.63	3.94	4.02	S62 CO	α
V66	5.67	5.19	7.17	> 8.5 *	S62 CO	α
A67	5.98	5.90	6.21	6.07	R63 CO	α
Q68	5.98	5.79	6.16	6.18	D64 CO	α
W69	> 7.0 *	6.71	> 8.5 *	> 8.5 *	D65 CO	α
L70	> 7.0 *	> 7.0 *	> 8.5 *	> 8.5 *	V66 CO	α
K71	6.86	6.44	7.66	7.42	A67 CO	α
W72	6.51	6.35	6.97	6.98	Q68 CO	α
A73	6.17	5.90	> 8.5 *	6.06	W69 CO	α

Residue	A93D-PNT Monomer log(PF) [a]	V112E-PNT Monomer log(PF) [a]	A93D-PNT Heterodimer log(PF) [a]	V112E-PNT Heterodimer log(PF) [a]	amide $^1H^N$ h-bond acceptor [b]	2º struct. [b]
E74	5.59	5.42		5.51	L70 CO	α
N75					K71 CO	α
E76	4.00	3.89	4.60	3.94	W72 CO	α
F77	4.39	4.30	7.12	4.28	A73 CO	
S78	4.44		5.70		A75 CO	
L79			5.24		Q74 CO	
R80						
P81	Proline	Proline	Proline	Proline		
I82						
D83						
S84						
N85						
T86					D83 CO	
F87					S84 CO	
E88						
M89	4.03		4.16	6.99		
N90				7.35	F77 CO	
G91					W61 CO	α
K92	4.39	4.13	4.64	7.19		α
A93	4.84	4.83	5.29	> 8.5 *	N90 CO	α
L94	5.23		6.68	> 8.5 *	N90 CO	α
L95	5.38		6.36	7.13	G91 CO	
L96	3.37	3.70	3.41	> 8.5 *	A93 CO	
L97	5.57		6.62	> 8.5 *	L94 CO	
T98	4.14	4.37		6.98		
K99						α
E100						α
D101	3.97	3.83	4.57	5.73	T98 CO	α
F102		4.74	7.45	7.67	T98 CO	α
R103					K99 CO	α
Y104			5.24	6.51	E100 CO	α
R105	4.31				D101 CO	α
S106		5.12	7.14	8.16	F102 CO	
P107	Proline	Proline	Proline	Proline		
H108						

Residue	A93D-PNT Monomer log(PF) [a]	V112E-PNT Monomer log(PF) [a]	A93D-PNT Heterodimer log(PF) [a]	V112E-PNT Heterodimer log(PF) [a]	amide $^{1}H^{N}$ h-bond acceptor [b]	2° struct. [b]
S109						
G110					S106 CO	α
D111			4.12			α
V112	3.18		5.29	3.46	S109 CO	α
L113	4.90		> 8.5 *	4.95	S109 CO	α
Y114	5.96		> 8.5 *	7.61	G110 CO	α
E115	5.86		5.58	6.22	D111 CO	α
L116	> 7.0 *		> 8.5 *	6.74	R112 CO	α
L117	> 7.0 *	> 7.0 *	> 8.5 *	> 8.5 *	L113 CO	α
Q118	5.84	6.01	6.95	5.89	Y114 CO	α
H119				5.65	E115 CO	α
I120	3.49	4.03	4.37		L116 CO	α
L121	3.01	3.71	3.35		L117 CO	α
K122			5.03		Q118 CO	α
Q123					H119 CO	
R124						
K125						

[a] Blank entries correspond to amides with unknown protection factors due to unassigned or overlapping signals in ^{15}N-HSQC spectra, or to amides that exchanged too rapidly for HX quantitation and thus have log(PF) values less than an estimated upper limit of ~ 3. Conversely, for amides with little exchange after 3 months in D_2O solution (*), the estimated lower limits of log(PF) values are > 7 (monomeric) and > 8.5 (heterodimeric). These are based on the highest log(PF) values measured for the corresponding species.

[b] Secondary structure and main chain carbonyl hydrogen bond acceptors for the amide $^{1}H^{N}$ are from an analysis of chains A and B of 1LKY.pdb using PDBsum (Laskowski et al., *Protein Sci*. 27: 129–134, 2018) and Vadar (Willard et al., *Nucleic Acids Res*. 31: 3316-3319, 2003), respectively. If multiple acceptors were identified, only the closest is indicated.

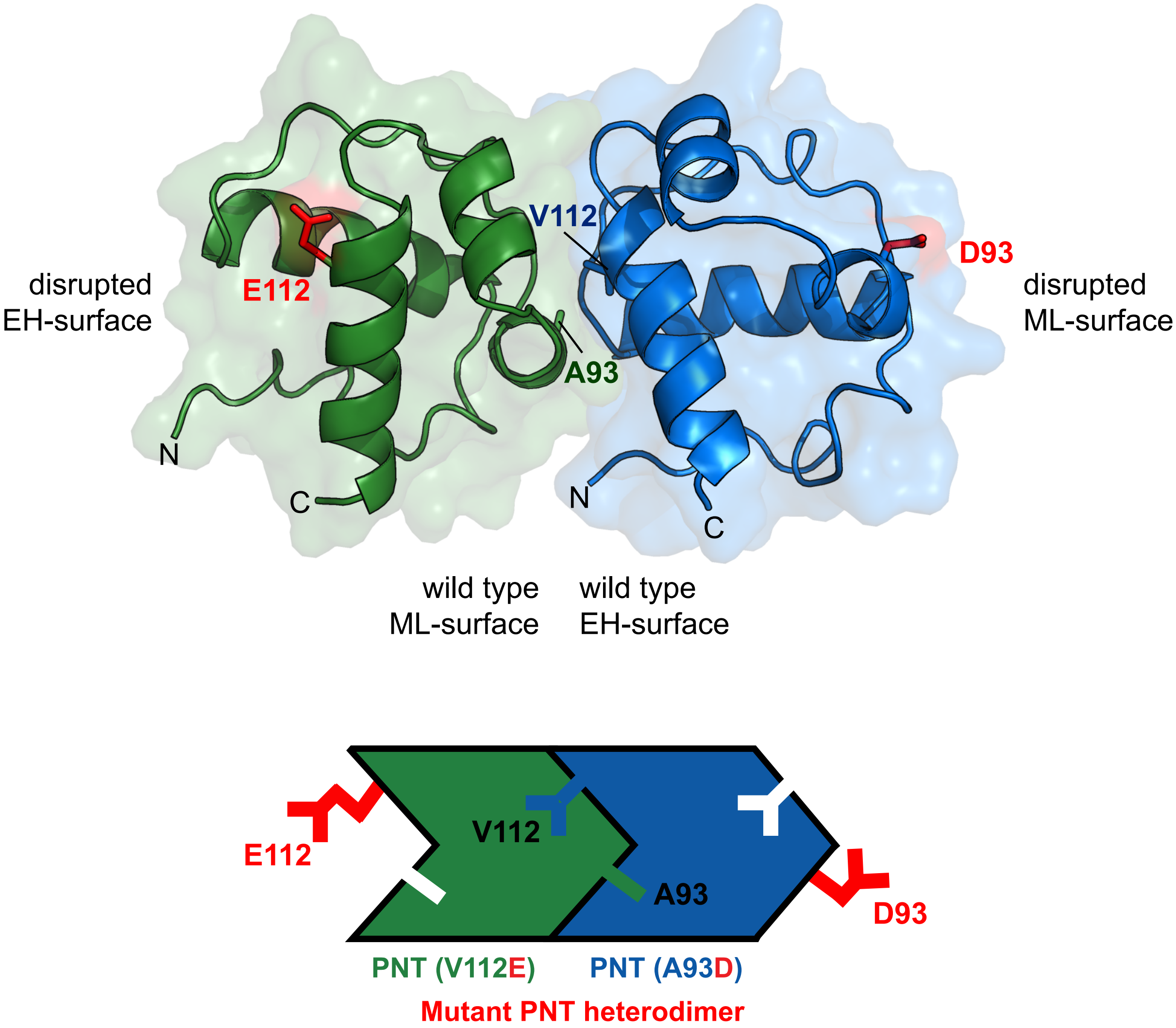

Figure S1. A heterodimer model of the ETV6 PNT domain polymer. The ETV6 PNT domain forms a head-to-tail polymer by self-associating through two interfaces, termed the ML- (mid-loop) and EH- (end-helix) surfaces. Substitution of Ala93 in the ML-surface or Val112 in the EH-surface with a charged residue renders the PNT domain monomeric. However, the two mutated proteins can still heterodimerize via their complementary wild type interfaces (Kim et al., *EMBO J.* 20: 4173–4182, 2001).

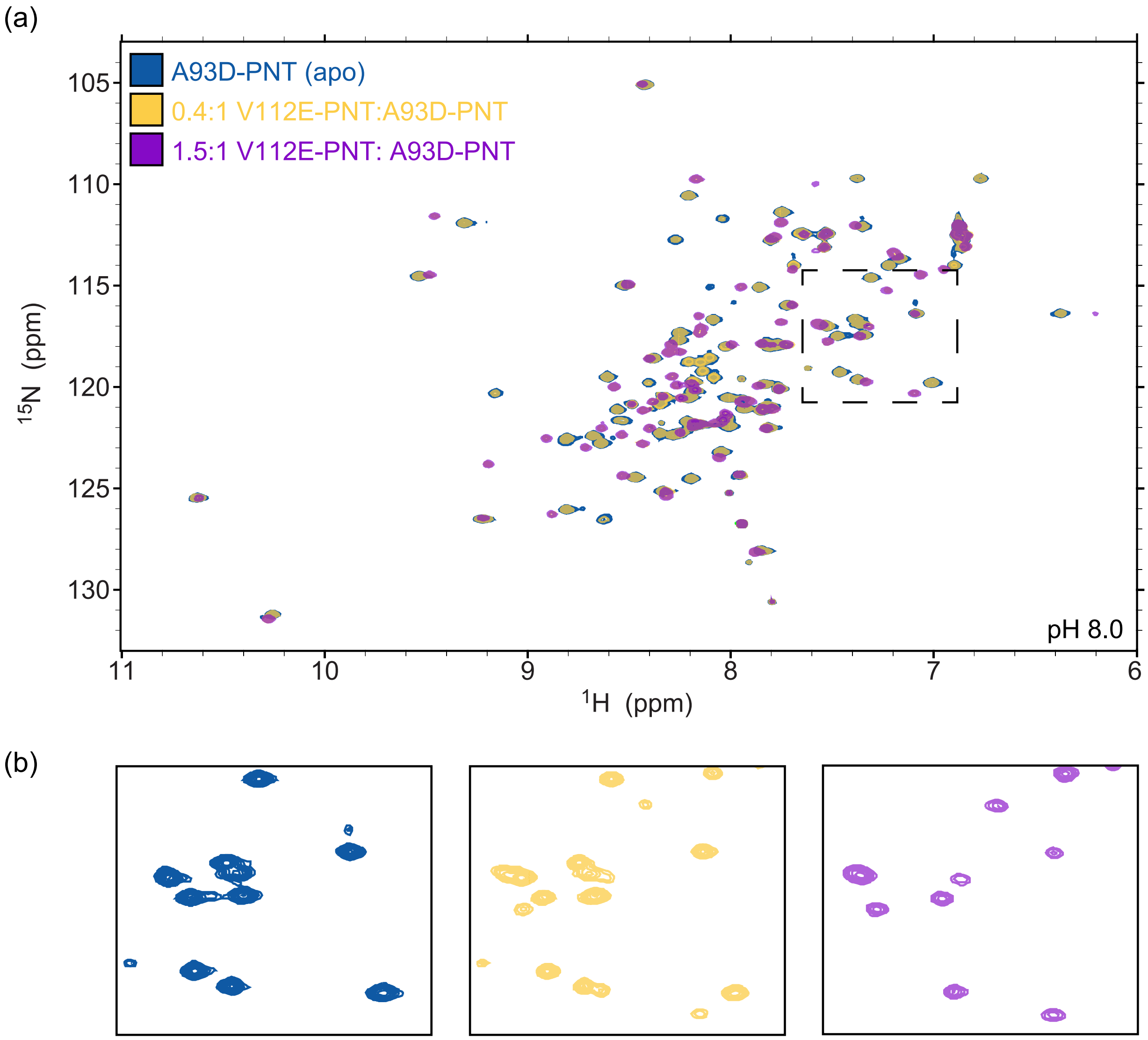

Figure S2. The A93D- and V112E-PNT domains bind in the slow exchange limit. (a) Overlaid ^{15}N-HSQC spectra showing the titration of unlabeled (NMR silent) V112E-PNT domain to ^{15}N-labeled A93D-PNT domain at 0:1 (blue), 0.4:1 (yellow) and 1.5:1 (purple) molar ratios. The region encompassed by the black dotted square is enlarged in (b). In the left panel of (b), only $^{1}H^{N}$-^{15}N peaks from the unbound A93D-PNT domain are present. In the middle panel, separate signals from amides in the unbound and bound protein are seen. In the right panel, only signals from the bound protein are detected. All spectra were collected in 20 mM MOPS, 50 mM NaCl, 0.5 mM EDTA and 5% D_2O at pH 8 and 25 °C.

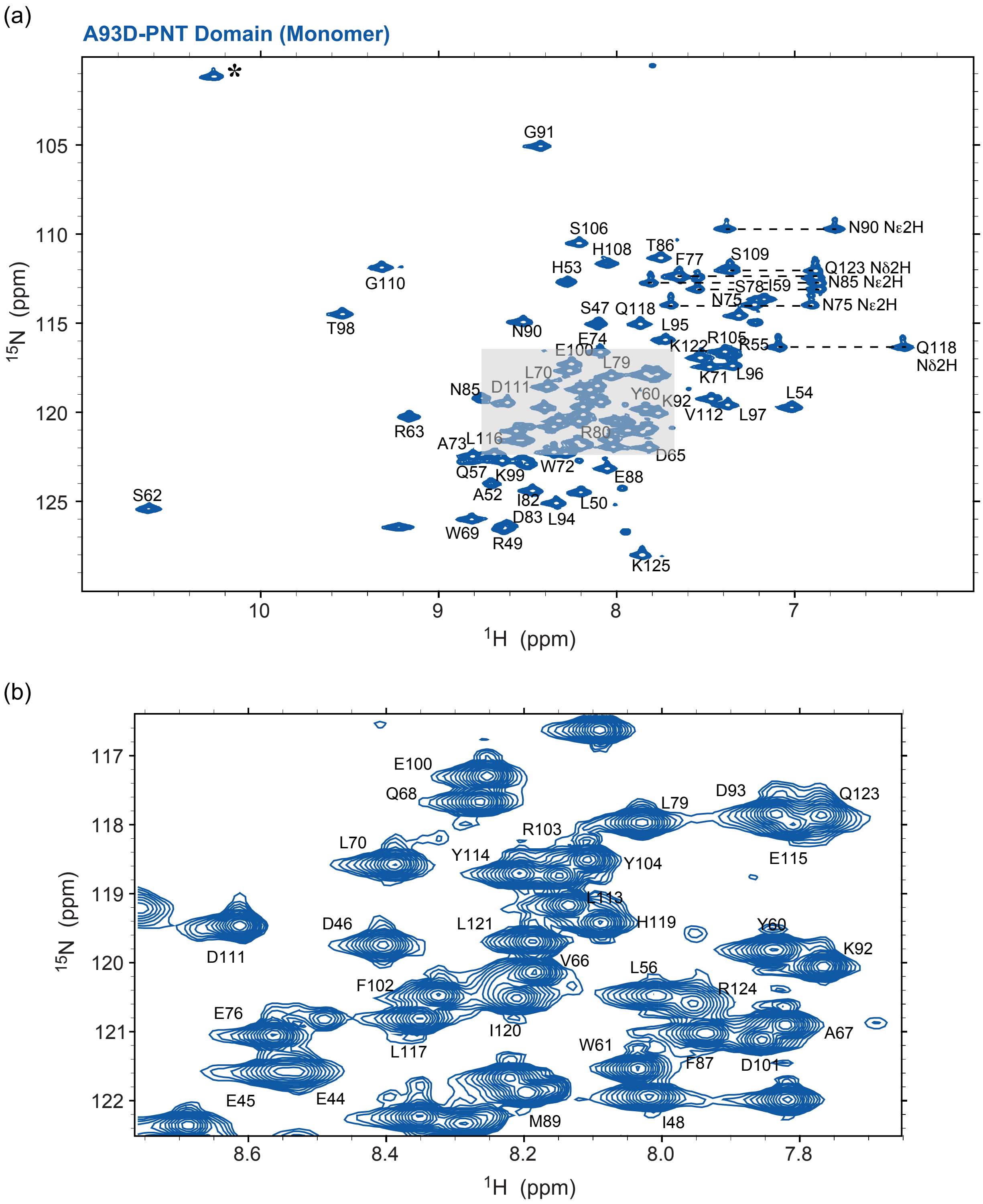

Figure S3. ^{15}N-HSQC spectrum of the monomeric A93D-PNT domain. (a) This spectrum was collected in 20 mM MOPS, 50 mM NaCl, 0.5 mM EDTA and 5% D_2O at pH 7.0 and 25 °C. Signals from the assigned mainchain amide $^{1}H^{N}$-^{15}N and several Gln and Asn sidechains are labeled, with the crowded central region (grey shading) enlarged for clarity in panel (b). An unassigned aliased peak from a tryptophan sidechain is identified with an asterisk. The chemical shift assignments have been deposited in the BMRB under accession number 50433.

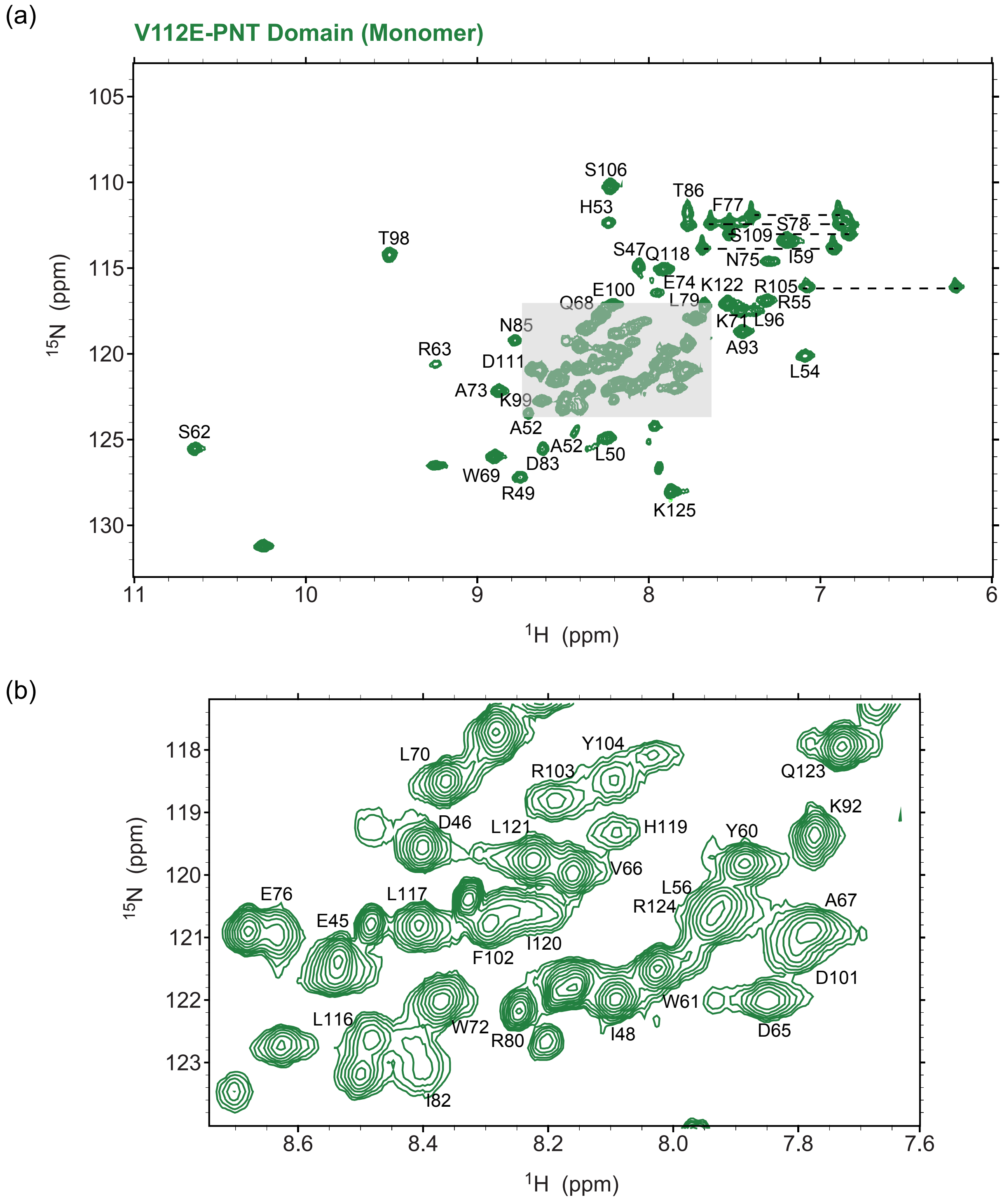

Figure S4. ^{15}N-HSQC spectrum of the monomeric V112E-PNT domain. (a) This spectrum was collected in 20 mM MOPS, 50 mM NaCl, 0.5 mM EDTA and 5% D_2O at pH 8.0 and 25 °C. The assigned mainchain amide $^{1}H^{N}$-^{15}N signals are labeled, with the crowded central region (grey shading) enlarged for clarity in panel (b). Horizontal dashed lines connect the unassigned signals from Asn and Gln sidechain amides. The chemical shift assignments have been deposited in the BMRB under accession number 50430.

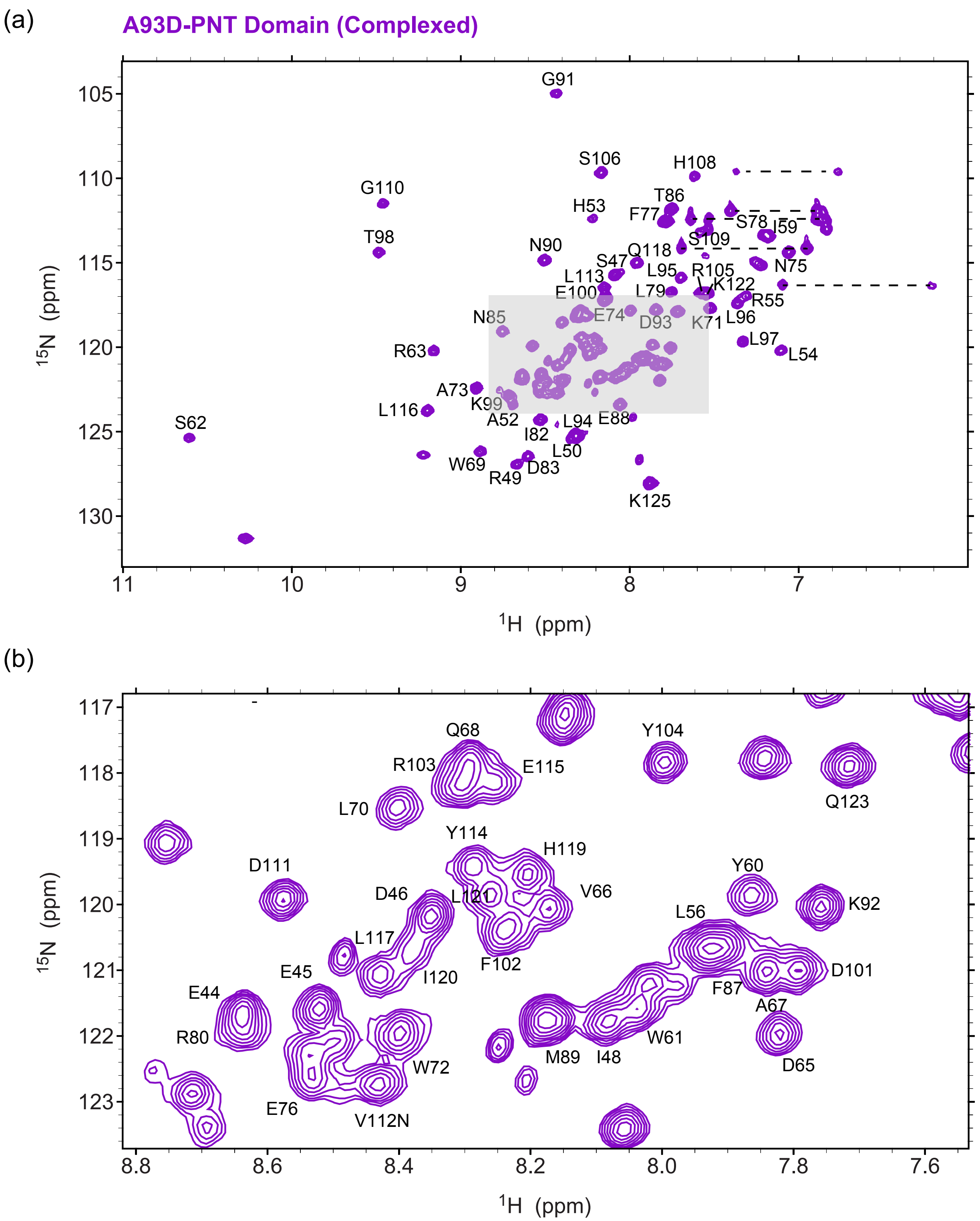

Figure S5. ^{15}N-HSQC spectrum of the ^{15}N-labeled A93D-PNT domain bound to the unlabeled V112E-PNT domain. (a) This spectrum was collected with a 1.1 molar excess of unlabeled protein in 20 mM MOPS, 50 mM NaCl, 0.5 mM EDTA and 5% D_2O at pH 7.0 and 25 °C. The assignments of mainchain amide $^{1}H^{N}$-^{15}N signals from the ^{15}N-labeled A93D PNT-domain are indicated, with the crowded central region (grey shading) enlarged for clarity in panel (b). The chemical shift assignments have been deposited in the BMRB under accession number 50432.

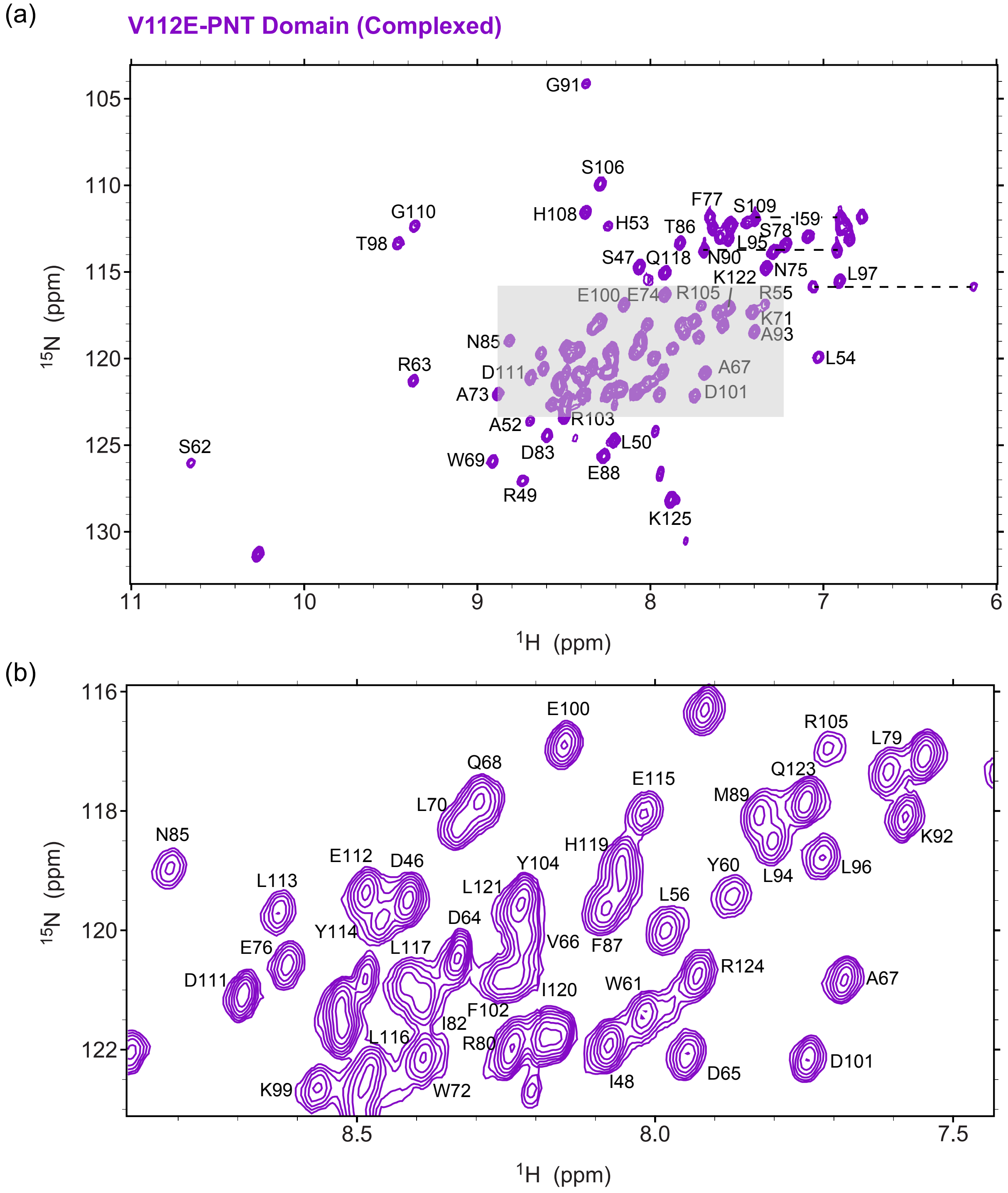

Figure S6. ^{15}N-HSQC spectrum of the ^{15}N-labeled V112E-PNT domain bound to the unlabeled A93D-PNT domain. (a) This spectrum was collected with a 1.1 molar excess of unlabeled protein in 20 mM MOPS, 50 mM NaCl, 0.5 mM EDTA and 5% D_2O at pH 7.5 and 25 °C. The assignments of mainchain amide $^{1}H^{N}$-^{15}N signals from the ^{15}N-labeled V112E-PNT domain are indicated, with the crowded central region (grey shading) enlarged for clarity in panel (b). The chemical shift assignments have been deposited in the BMRB under accession number 50431.

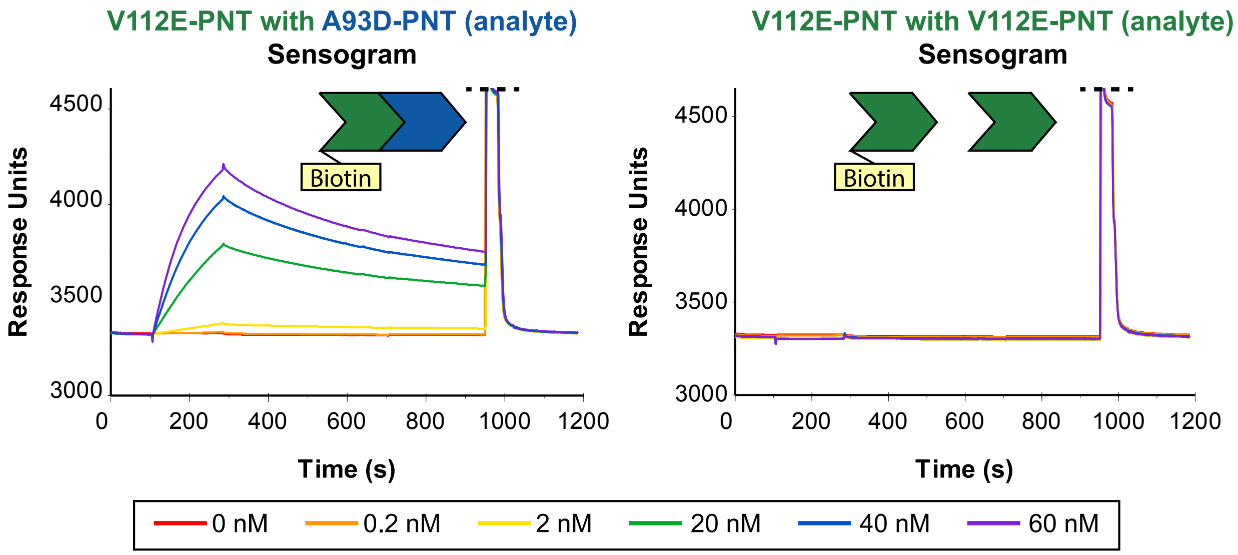

Figure S7. SPR provides a reliable measure of the A93D- and V112E-PNT domain interactions. Shown are Biacore X100 SPR sensograms for control experiments with either the A93D-PNT domain (left, blue cartoon shape) or V112E-PNT domain (right, green) analyte passed over the biotinylated V112E-PNT domain (green) ligand immobilized on a streptavidin chip. Different concentrations of analyte were run over the chip for 300 sec, followed by buffer only to allow dissociation. A 30 second regeneration wash with 0.2% SDS produced the response unit (RU) spike at ~ 1000 seconds (truncated at the dashed line) and returned the baseline back to its starting value. Fitting of these concentration dependence response curves demonstrated heterodimer formation between the A93D- and V112E-PNT domains via their wild type interfaces with a K_D value of 7.5 nM. In contrast, the V112E-PNT domain did not measurably self-associate.

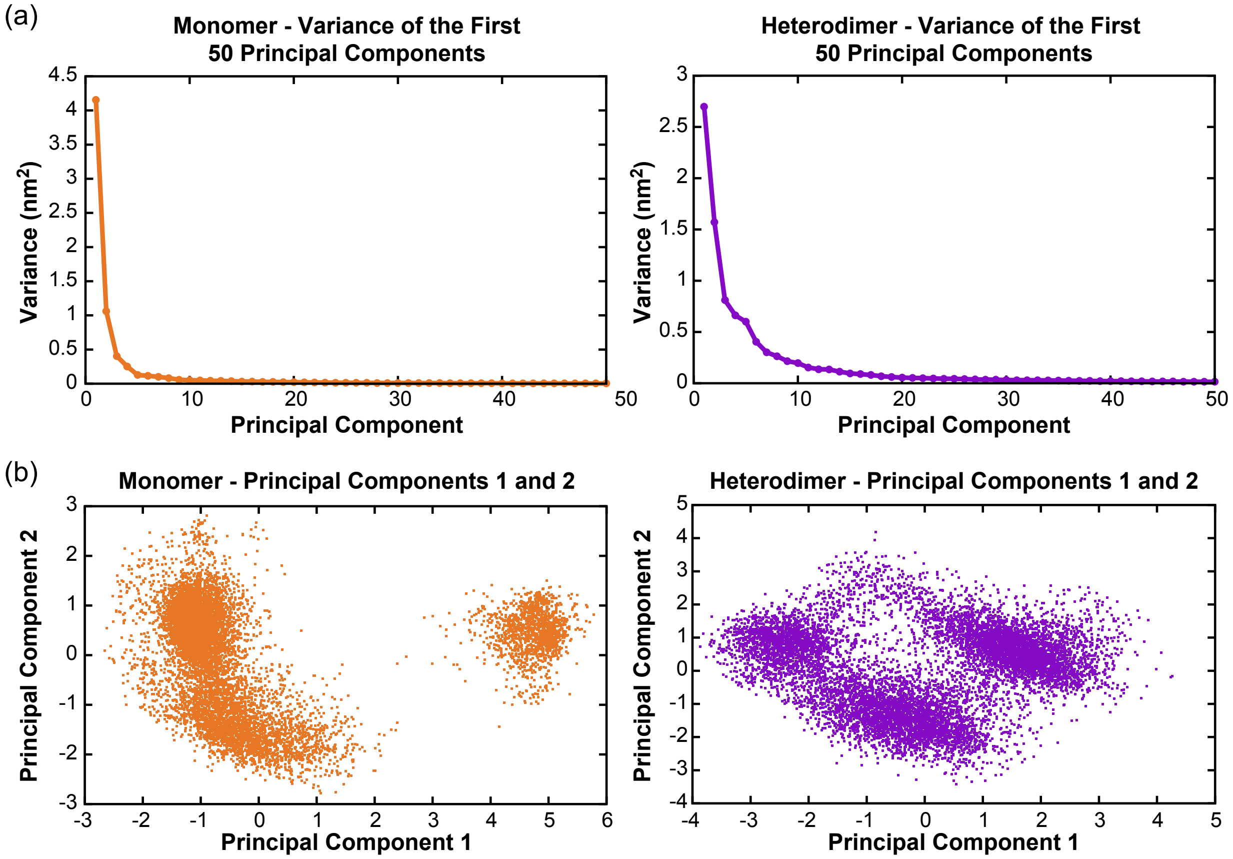

Figure S8. Principal component analysis of the monomer and heterodimer trajectories. (a) Shown are the variance (eigenvalues) for the first 50 principal components (eigenvectors) extracted from the 1 μs MD simulations of the monomeric A93D-V112E-PNT domain (left), and a heterodimer of the A93D-PNT domain and V112R-PNT domain from PDB: 1LKY (right). (b) Projection of the trajectories onto the first two principal components (PCs). Many combinations of PC1 and PC2 are accessed, and thus the MD simulations provided a good sampling of the conformational space available to the ETV6 PNT domain. Notably, a large proportion of the variance is contained in the first two PCs of the monomer (71%) and in the first four PCs of the heterodimer (56%). All of these modes correspond to flexing of the protein termini (Movies S1-S4). PC1 also includes the large change in the N-terminal 10 residues occurring around 150 ns in the trajectory of the monomer. This is reflected in the smaller population at positive PC1 corresponding to the initial position of the N-terminal segment, whereas the major population at negative PC1 corresponds to its flipped state. PC2 is more evenly sampled, but appears to exhibit two preferred conformations at 0.8 and -1.3. The low variance in the higher PCs and the low RMSD values of the protein core, with the flexible termini, both illustrate the overall rigidity of the ETV6 PNT domain.

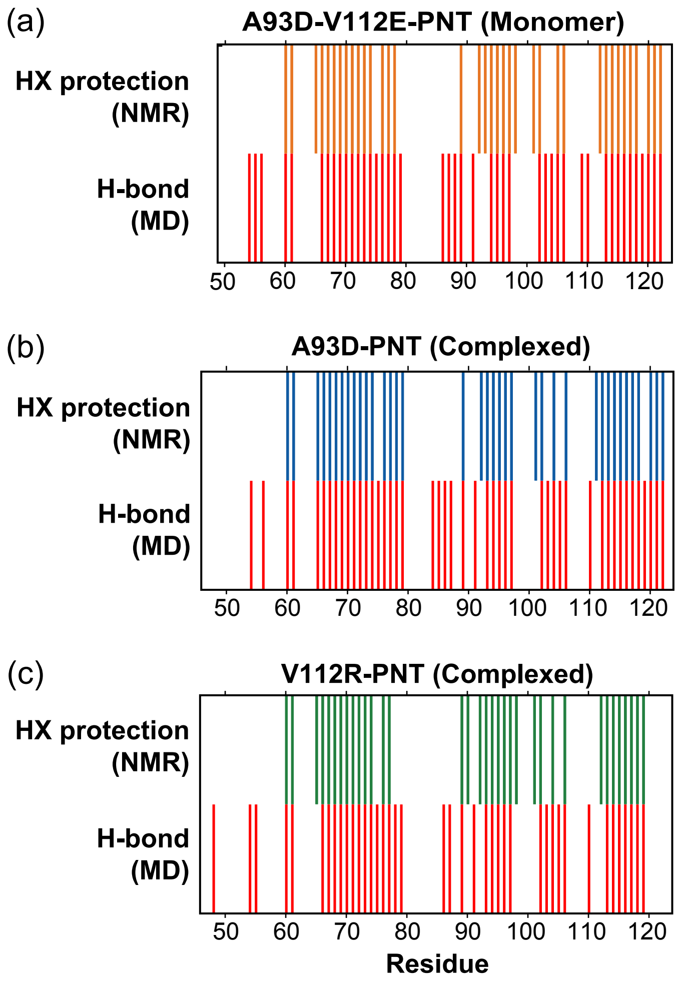

Figure S9. Correlation between amide hydrogen bonding during MD simulations and experimentally measured HX protection. The red bars identify amides participating in backbone hydrogen bonds with an average length ≤ 3.5 Å over the 1 μs MD simulations of (a) the monomeric A93D-V112E-PNT domain, and a heterodimer of the (b) A93D-PNT domain and (c) V112R-PNT domain from PDB: 1LKY. The orange bars in (a) also identify amides in either the monomeric A93D-PNT domain or V112R-PNT domain showing HX protection measurable by NMR spectroscopy (i.e. log(PF) > 3, Table S3). Similarly the blue bars in (b) and the green bars in (c) identify protected amides in the complexed forms of the A93D-PNT domain and V112E-PNT domain, respectively. Missing data correspond to amides with unknown protection factors due to unassigned or overlapping signals in ^{15}N-HSQC spectra, or to amides that exchanged too rapidly for HX quantitation and thus have log(PF) values less than an estimated upper limit of ~ 3.

Movies S1-S4. Principal component analysis of the monomer and heterodimer trajectories. The movies show the motions about the first two principle components (PC1 and PC2) extracted from the 1 μs MD simulations of the monomeric A93D-V112E-PNT domain (Movies S1 and S2, respectively, with the N-terminus upper left) and the heterodimeric A93D- and V112R-PNT domains (Movies S3 and S4, respectively, with the V112R-PNT domain on the left).
